# Interfering cellular lactate homeostasis overcomes Taxol resistance of breast cancer cells through the microRNA-124-mediated lactate transporter (MCT1) inhibition

**DOI:** 10.1186/s12935-019-0904-0

**Published:** 2019-07-24

**Authors:** Lu Hou, Yi Zhao, Guo-qing Song, Ying-han Ma, Xiao-hu Jin, Si-li Jin, Yi-han Fang, Yi-chong Chen

**Affiliations:** The 1st Breast Surgical Department, Affiliated Shengjing Hospital of China Medical University, Shenyang, 110004 Liaoning China

**Keywords:** Breast cancer therapy, MicroRNA-124, Taxol resistance, Lactate homeostasis, MCT1 inhibitor

## Abstract

**Background:**

Breast cancer, the most common invasive cancer of women, is a malignant neoplasm and the second main cause of cancer death. Resistance to paclitaxel (Taxol), one of the frequently used chemotherapy agents for breast cancer, presents a major clinical challenge. Recent studies revealed that metabolic alterations of cancer cells play important roles in chemo-resistance.

**Materials and methods:**

In this study, Human breast cancer cells, BT474, SKBR3 and MCF7 were used to study the causal relationship between the lactate exporter, MCT1 (SLC16A1)-modulated glucose metabolism and Taxol resistance of breast cancer cells. Taxol resistant breast cancer cells were established. The intracellular lactate and extracellular lactate levels as well glucose uptake and oxygen consumption were measured. MicroRNA-124 expressions were detected by qRT-PCR from both breast cancer patient samples and breast cancer cells. Target of miR-124 was predicted and verified by Western blot and luciferase assay. An xenograft mice model was established and evaluated for the in vivo tumor therapeutic effects of MCT1 inhibitor plus microRNA-124 treatments.

**Results:**

Low toxic Taxol treatments promoted cellular glucose metabolism and intracellular lactate accumulation with upregulated lactate dehydrogenase-A (LDHA) and MCT1 expressions. By establishing Taxol resistant breast cancer cell line, we found Taxol resistant cells exhibit upregulated LDHA and MCT1 expressions. Furthermore, glucose consumption, lactate production and intracellular ATP were elevated in Taxol resistant MCF7 cells compared with their parental cells. The miR-124, a tumor suppressive miRNA, was significantly downregulated in Taxol resistant cells. Luciferase assay and q-RT-PCR showed MCT1 is a direct target of miR-124 in both breast cancer cell lines and patient specimens. Moreover, co-treatment of breast cancer cells with either MCT1 inhibitor or miR-124 plus Taxol led to synergistically cytotoxic effects. Importantly, based on in vitro and in vivo results, inhibition of MCT1 significantly sensitized Taxol resistant cells. Finally, rescue experiments showed restoration of MCT1 in miR-124 overexpressing cells promoted Taxol resistance.

**Conclusions:**

This study reveals a possible role of miRNA-214-mediated Taxol resistance, contributing to identify novel therapeutic targets against chemoresistant breast cancers.

## Background

Breast cancer is one of the most frequently diagnosed cancers among women [1, 2]. In addition to surgery, adjuvant systemic therapies including endocrine therapy, anti-HER2 therapy, and chemotherapy have been reported to effectively reduce the risk of local and distant recurrence [3]. Taxol (paclitaxel) is an essential chemotherapeutic agent for the treatment of multiple types of tumor, including breast cancer through disrupting the dynamic equilibrium between tubulin dimers and their polymerized form [4]. Yet, a significant percentage of breast cancer patients fail to respond to initial chemotherapy due to development of resistance during the treatment with Taxol, limiting the clinical efficacy of Taxol [4, 5]. Thus, it is imperative to investigate novel strategies which could overcome chemoresistance in cancers.

Warburg first postulated that cancer cells, in contrast to normal cells rely on glycolysis than oxidative phosphorylation to produce ATP for proliferation [6]. Moreover, in cancer cells, pyruvate produced from glycolysis was converted to lactate by lactate dehydrogenase A (LDHA) rather than acetyl-CoA [7]. This phenomenon is called the “Warburg effect” [8]. Dysregulated glycolytic flux leads to high levels of lactate, which is exported by transporters (MCTs) on the plasma membrane [9]. The MCT (monocarboxylic acid solute transporters) family includes 14 members, and MCT1-4 have been reported to mediate bi-directional transport of lactate [10]. Studies demonstrated that MCT1 expressions positively correlate with glycolytic metabolism and malignancy in breast cancer [11]. Moreover, MCT1 inhibition could suppress growth and induce cell death of breast cancers through disruption of glycolysis and glutathione synthesis [12], suggesting blocking the MCT1-mediated glycolysis might contribute to improvement of chemotherapeutic effects.

MicroRNAs (miRNAs) are a group of small, noncoding RNAs (22-25 nucleotides) that play important roles in diverse cancer processes, such as tumorigenesis, development, differentiation, invasion, migration and cell death [13, 14]. MiRNAs lead to post-transcriptional silencing of their target mRNAs through complementary binding to the 3′ untranslated region (UTR) of mRNA targets [14, 15]. Multiple studies reveal that miR-124 negatively regulates carcinogenesis with the observations that miR-124 expression level is significantly downregulated in oral squamous cell carcinoma (OSCC) [16], glioma [17], colon cancer [18], lung cancer [19], hepatocellular carcinoma (HCC) [20], breast cancer [12] and bladder cancer [21]. However, the molecular mechanisms underlying the miR-124-regulated malignant phenotype of breast cancer cells are still under investigation. In this study, roles of the axis miR-124-MCT1-Taxol sensitivity of breast cancer will be studied. We will predict and verify the direct target of miR-124 in breast cancer cells and patient specimens. Furthermore, the synergistic effects of the combination of MCT1 inhibitor and Taxol treatments on breast cancer will be investigated in vitro and in vivo.

## Materials and methods

### Cell culture and tumor specimens

Human breast cancer cell lines BT474, MCF7, SKBR3 and MDA-MB-231 were purchased from the American Type Culture Collection (Manassas, VA, USA). All cells were cultured in DMEM/F12 (Dulbecco’s Modified Eagle Medium: Nutrient Mixture F-12) medium and maintained at 37 °C in a 5% CO_2_ humidified incubator. A cohort of 60 human primary breast cancer specimens during surgery, collected during the surgery at the Affiliated Shengjing Hospital of China Medical University, China, were examined in this study. All protocols concerning the use of patient specimens were approved by the Medical Ethics Committee of Affiliated Shengjing Hospital of China Medical University. The informed consent was signed by all the patients involved in the study. All breast cancer specimens were from female patients classified according to current World Health Organization criteria. The ages of adult patients were from 25 to 44 years.

### Antibodies and reagents

Paclitaxel and SR13800 were purchased from Sigma-Aldrich (St. Louis, MO, USA). Antibodies against β-actin (#4970) and LDHA (#3582) were purchased from Cell Signaling (Beverly, MA, USA). Monoclonal mouse antibody against MCT1 was purchased from ThermoFisher Scientific (#MA5-18288, Waltham, MA, USA). Control siRNA and MCT1 siRNA (5′-GAGGAUGAAUCACUAAGUA-3′) were synthesized by Ribobio (Guangzhou, China).

### MiRNAs transfection and siRNA knockdown

MiRNAs and siRNA transfections were performed using Lipofectamine RNAiMAX reagents (ThermoFisher Scientific, Waltham, MA, USA) according to the manufacturer’s instructions. Briefly, 2 × 10^5^ cells/well were plated in 6-well plate for overnight. After refreshing medium, cells were transfected with miRNAs (25 nM) or siRNA (50 nM) for 48 h followed by downstream experiments.

### Glucose consumption and lactate assays

The glucose consumption and lactate product assays were performed as previously reported [22, 23] using the Glucose Uptake Assay Kit (Colorimetric) (ab136955) and L-Lactate Assay Kit (Colorimetric) (ab65331) from Abcam (Cambridge, UK) according to the manufacturer’s instructions. The intracellular and extracellular lactate were detected using an absorbance-based assay kit (Biovision, Milpitas, CA, USA) according to the previous report [12]. All experiments were performed in triplicate and repeated three times.

### ATP/ADP ratio assay

Intracellular ATP/ADP ratio was determined with an ADP/ATP Ratio Assay kit (Cat. No. ab65313, Abcam) according to the manufacture’s instruction. Briefly, 1x10^4^ cells/well were plated in 96 well plates for overnight. The luminescence was measured in a luminometer. ATP/ADP ratio was calculated as 1 − [Data D − Data C]/[Data B − Data A]. Data D: sample signal ~ 2 min after addition of 10 μL 1× ADP Converting Enzyme to cells; Data C: sample signal prior addition of 1× ADP Converting Enzyme to cells; Data B: sample signal ~ 2 min after addition of cells to reaction mix; Data A: background signal of reaction mix. All experiments were performed in triplicate and repeated three times.

### Oxygen consumption rate (OCR)

OCR was measured using the Seahorse Bioscience XF96 Analyzer according to previous reports [12]. Final concentration of agents used was oligomycin 1 mmol/L (Calbiochem), carbonyl cyanide 4-(trifluoromethoxy) phenylhydrazone (FCCP) 300 nmol/L (Sigma), and rotenone 100 nmol/L (Sigma). Protein concentration of each sample was measured using Bradford method to normalize OCR results. All experiments were performed in triplicate and repeated three times.

### Measurement of cell viability

Cell viability was measured by MTT assay (MD Millipore, Billerica, MA, USA). Briefly, cells were cultured at 3 × 10^4^ cells/well in a 96-well culture plates for overnight. Then 3-(4,5-dimethylthiazol-2-yl)-2,5-diphenyltetrazolium bromide (MTT) at a concentration of 5 mg/mL was added to each well at 10 μL/100 μL followed by incubation at 37  °C for 2 h in a cell culture incubator. The medium was replaced with 100 μL dimethyl sulfoxide (DMSO), and the absorbance for each well was measured at 570 nm using a scanning multiwell spectrometer (Bio-Tek instruments, Inc., Burlington, VT, USA). All experiments were performed in triplicate and repeated three times.

### RNA isolation and real-time PCR

Total RNA was extracted using a TRIzol Reagent (Invitrogen, Carlsbad, CA) according to the manufacturer’s instruction and subsequently treated with RNase-free DNase I (Fermentas, San Diego, CA, USA). For detection of mRNA expressions, cDNA synthesis was performed from 1 µg total RNA using a SuperScript First-Standard Synthesis system for RT-qPCR (Invitrogen Life Technologies). Real-time PCR was performed by an ABI PRISM 7900 Sequence Detection System (Applied Biosystems, Foster City, CA, USA) using Quanti-Tect SYBR Green PCR mixture (Qiagen, Hilden, Germany). The qPCR primers were: MCT1: forward: 5′-GTGGCTCAGCTCCGTATTGT-3, reverse: 5′-GAGCCGACCTAAAAGTGGTG-3′; LDHA: forward: 5′-TGGAGTGGAATGAATGTTGC-3′, reverse: 5′-ATAGCCCAGGATGTGTAGCC-3′; and β-actin: forward: 5′-AGGCACCAGGGCGTGAT-3′, reverse: 5′-GCCCACATAGGAATCCTTCTGAC-3′. For detection of miRNAs, reverse transcription reaction was performed using the TaqMan Advanced miRNA cDNA Synthesis Kit (ThermoFisher Scientific, Waltham, MA, USA) according to the instructions. qPCR was performed by ABI PRISM 7900 Sequence Detection System. The reactions were incubated in a 96-well optical plate at 95 °C for 10 min, followed by 40 cycles at 95 °C for 15 s and 60 °C for 1 min. RNU6 was used as internal control. Relative expressions were calculated by the 2^−△△CT^ method. All reactions were performed in triplicate.

### Luciferase assay

Luciferase reporter assay was done by transient co-transfection of pMIR-REPORT vector (Ambion) containing wild type or mutant 3′UTR of human MCT1 containing the putative miR-124 binding site and miR-124 mimic or control mimic into 293T cells in 24-well plates using Lipofectamine 2000 (Invitrogen) according to the manufacturer’s instructions. For luciferase reporter assay, 48 h after transfection, luciferase activity was measured using the Dual-Luciferase Reporter Assay System (Promega). Luciferase activity was read by SpectraMax M5 (Molecular Devices, Sunnyvale, CA). Experiments were performed in triplicate and repeated three times.

### Western blot analysis

Cells were lysed using RIPA buffer (#89900, ThermoFisher, Waltham, MA, USA) with 1× protease inhibitor mix (GE Healthcare, Piscataway, NJ, USA) and incubated on ice for 15 min. After centrifugation at 14,000*g*, the supernatant was saved. Protein concentration was determined by the Bradford assay. Equal amount of proteins was mixed with 2× sample buffer (Bio-Rad), followed by heating for 3 min. Samples were resolved by electrophoresis were transferred to PVDF membrane and blocked with 5% BSA for 1 h at room temperature. Membranes were then probed with primary antibodies at 1:1000 concentrations at 4 °C for overnight. After washing, membranes were incubated with secondary antibodies at room temperatures for 1 h. Protein expression was detected by ECL Plus Western blotting reagents (GE Healthcare) with appropriate horseradish peroxidase-conjugated secondary antibody. Images were taken using LAS-3000 Imaging System from Fuji. All experiments were repeated three times.

### Xenograft experiments

Animal experiments in this study were approved by the Affiliated Shengjing Hospital of China Medical University, China. A total of 32 female BALB/c nude mice were used. Xenograft tumors were established via injection of 1 × 10^6^ MCF7 Taxol resistant cells into the fat pad of mammary gland of 6-week old mice. After tumors reached 5 mm in diameter, the tumor-bearing mice were randomly assigned to four groups: Control (saline, n = 8), Taxol alone (n = 8), Taxol plus SR13800 (MCT1 inhibitor) (n = 8) and Taxol plus miR-124 mimic (n = 8). Tumor diameters were measured with calipers twice per week for 4 weeks. The tumor volumes were calculated with the following formula: volume (mm^3^ = width × length/2), where width and length are the minor and major diameters, respectively.

### Statistical analysis

Statistical analysis was performed using GraphPad Prism 5.0 software. Statistical significance between two groups was analyzed by Two-tailed Student’s t-test and significance among three or more groups was analyzed by ANOVA followed by post hoc analysis. *p* values < 0.05 were considered significant.

## Results

### Taxol treatments induce lactate secretion and expressions of MCT1 and LDHA

**Fig. 1:**
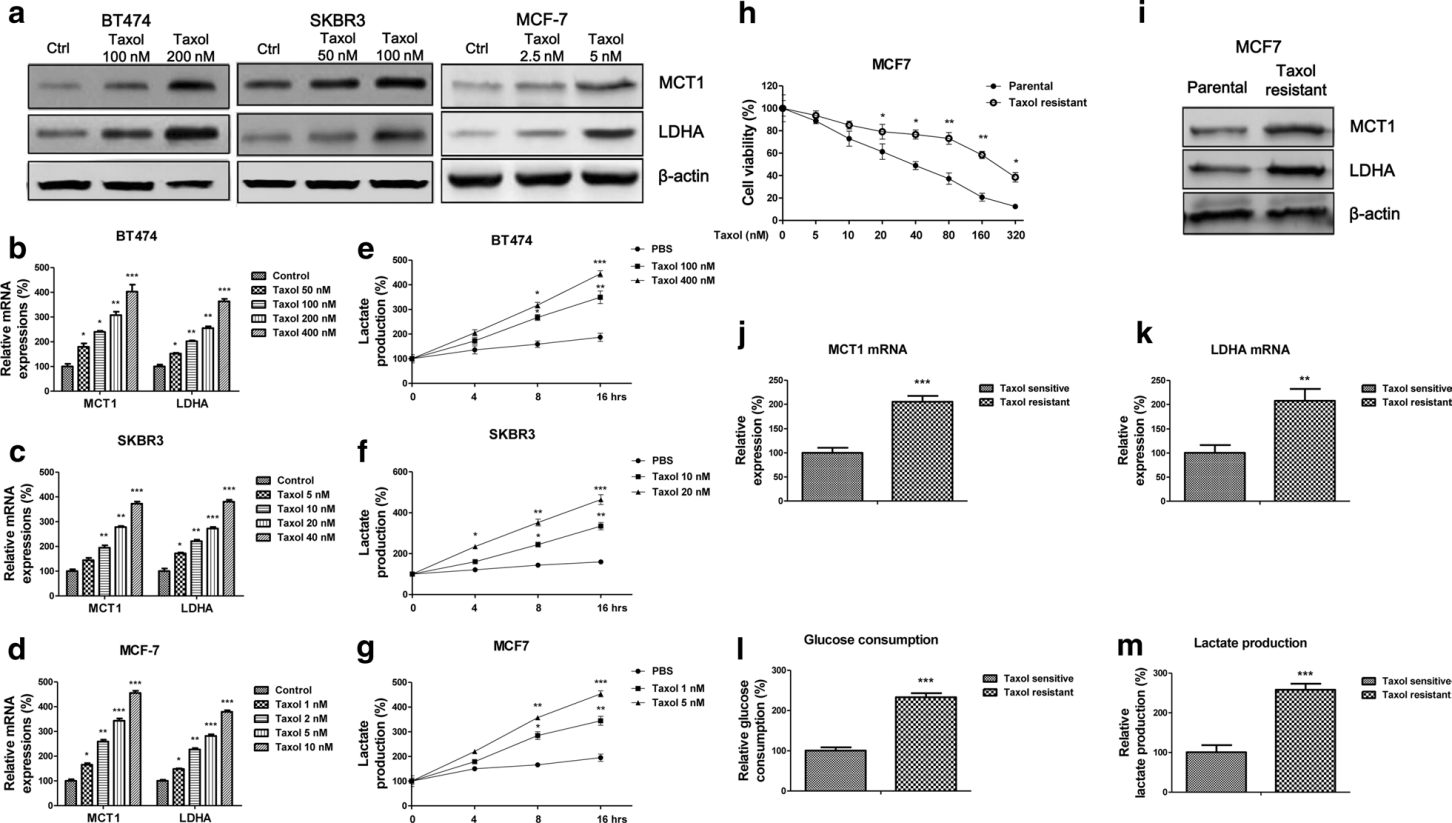
Lactate homeostasis is positively correlated with Taxol resistance of breast cancer cells. **a** BT474, SKBR3 and MCf7 cells were treated with indicated concentrations for 48 h, the protein levels and (**b**–**d**) mRNA levels of LHDA and MCT1 were detected by Western blot and qRT-PCR, respectively. **e**–**g** Lactate productions of BT474, SKBR3 and MCF7 cells were measured under indicated Taxol treatments. **h** Cell viability assays were performed in MCF7 parental and Taxol resistant cells under 0, 5, 10, 20, 40, 80, 160 and 320 nM Taxol treatments for 48 h. **i** Protein and (**j**, **k**) mRNA expressions of MCT1 and LDHA were detected in MCF7 parental and Taxol resistant cells by Western blot and qRT-PCR, respectively. β-actin is a loading control. **l** Glucose consumption and (M) lactate production were measured in MCF7 parental and Taxol resistant cells. *p < 0.05; **p < 0.01; ***p < 0.001

It was known that Taxol sensitivity of cancers was tightly correlated with cellular metabolism [24]. Thus, we started to assess the effects of Taxol treatments on the intracellular lactate production and extracellular lactate secretion, both are indicators of altered glucose metabolism. Human breast cancer cell lines, BT474, SKBR3 and MCF7 were treated with variant concentrations of Taxol for 48 h, since they have different sensitivities for Taxol (the IC50 was different). As we expected, the LDHA and MCT-1 protein expressions (Fig. 1a) and mRNA expressions (Fig. 1b–d) were significantly upregulated. Moreover, the extracellular lactate amounts were increased under Taxol treatments (Fig. 1e–g), suggesting Taxol treatments induced lactate production and secretion in breast cancer cells.

### Taxol resistant breast cancer cells show upregulated glycolysis, MCT1 and LDHA expressions

To further investigate the correlation between Taxol and lactate production/secretion, we established Taxol resistant breast cancer cell line originating from MCF7 parental cells by selecting survival cells under gradually elevated Taxol treatments. Results in Fig. 1h demonstrated Taxol resistant MCF7 cells could tolerant higher concentrations of Taxol compared with their parental cells. The IC50 of Taxol resistant cells was increased to 235.4 nM, which is approximately six-fold of that from parental cells (38.5 nM). Consequently, we compared the protein and mRNA expressions of MCT1 and LDHA from Taxol parental and resistant cells. We found both MCT1 and LDHA were significantly upregulated in Taxol resistant cells (Fig. 1i–k). To evaluate the glycolysis rates between MCF7 parental and Taxol resistant cells, the glucose consumption and lactate production were measured. Expectedly, glucose consumption lactate product assays consistently increased in Taxol resistant cells (Fig. 1l–m). In general, promoted glycolysis is positively correlated with Taxol resistance.

### Inhibition of MCT1 derails lactate homeostasis and glycolysis of Taxol resistant cells

**Fig. 2:**
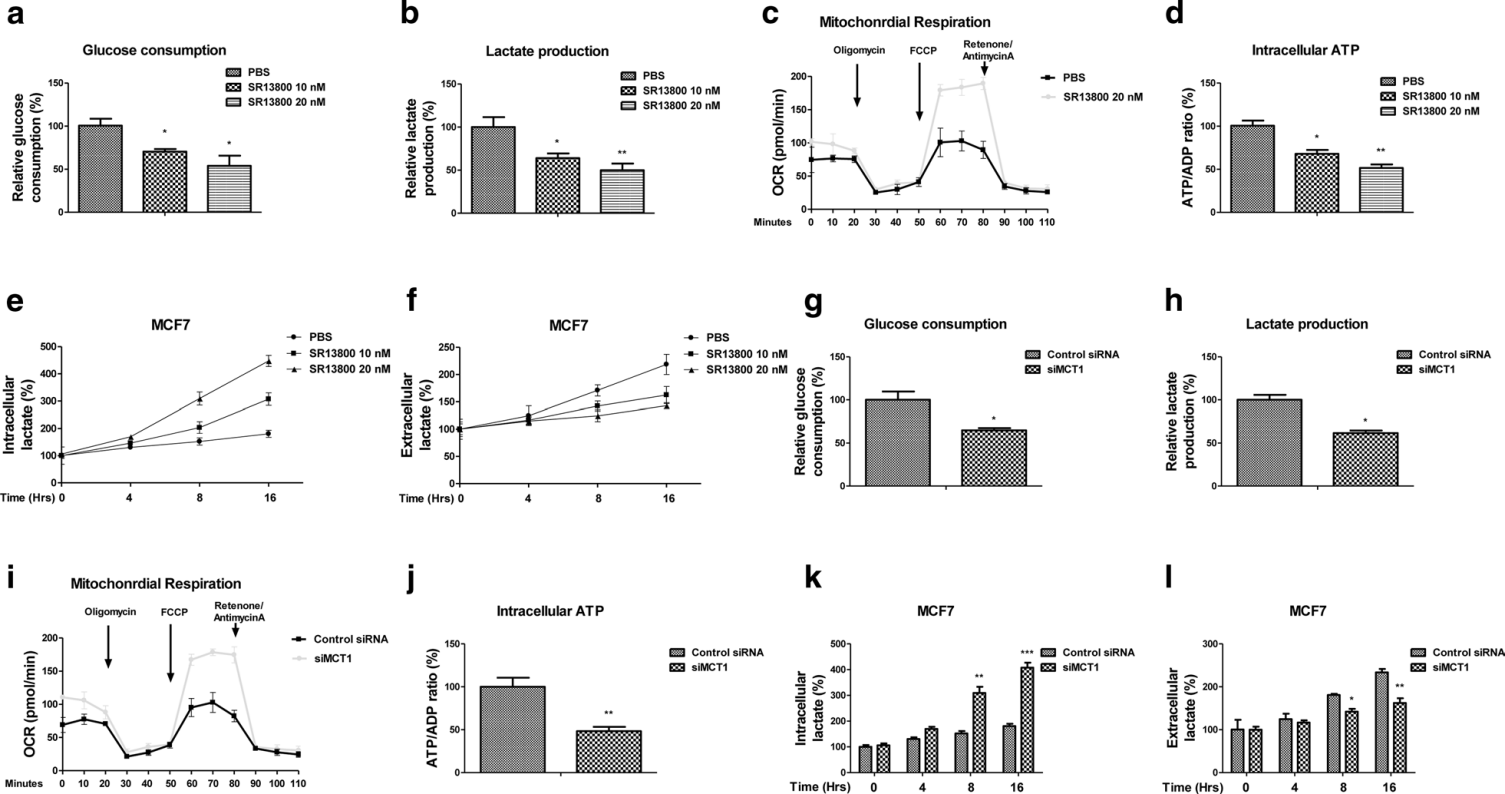
Inhibition of MCT1 renders a metabolic-switch phenotype. MCF7 cells were treated with SR13800 at 0, 10 or 20 nM for 48 h, the (**a**) glucose uptake, (**b**) lactate production, (**c**) OCR, (**d**) cellular ATP/ADP ratio, (**e**) intracellular lactate and (**f**) extracellular lactate were measured. **g** MCF7 cells were transfected with control siRNA or siMCT1 for 48 h, the glucose uptake, (**h**) lactate production, (**i**) OCR, (**j**) cellular ATP/ADP ratio, (**k**) intracellular lactate and (**l**) extracellular lactate were measured. *p < 0.05; **p < 0.01; ***p < 0.001

We next evaluated whether blocking lactate export through inhibiting MCT1 could impair homeostasis of glucose metabolism of breast cancer cells. To achieve this, we treated MCF7 cells with SR13800, a cell-permeable inhibitor of MCT1 [12]. Expectedly, glucose consumption and lactate production were significantly suppressed by SR13800 treatments (Fig. 2a, b). Interestingly, we found the oxygen consumption rate was increased by blocking MCT1 (Fig. 2c), suggesting MCT1 inhibition triggered an invert “Warburg effect”. In addition, the intracellular ATP/ADP ratio was significantly decreased under MCT1 inhibition (Fig. 2d). The intracellular lactate levels were increased at 8 and 16 h under SR13800 treatments (Fig. 2e), while the lactate export were decreased accordingly by blocking MCT1 (Fig. 2f), demonstrating a dysregulated lactate cellular accumulation under MCT1 inhibition. To further evaluate the glycolysis inhibitory effects by MCT1 inhibition, we knocked down endogenous MCT1 expressions in MCF7 cells. Consistent results showed knockdown MCT1 significantly suppressed glucose consumption (Fig. 2g) and lactate production (Fig. 2h), increased OCR (Fig. 2i) and decreased cellular ATP (Fig. 2j). Moreover, MCF7 cells with MCT1 knockdown displayed increased cellular lactate accumulation (Fig. 2k, l). Taken together, the above results demonstrated blocking MCT1 impaired lactate homeostasis of breast cancer cells.

### MiR-124 associates with Taxol sensitivity of breast cancer cells and directly targets MCT1

**Fig. 3:**
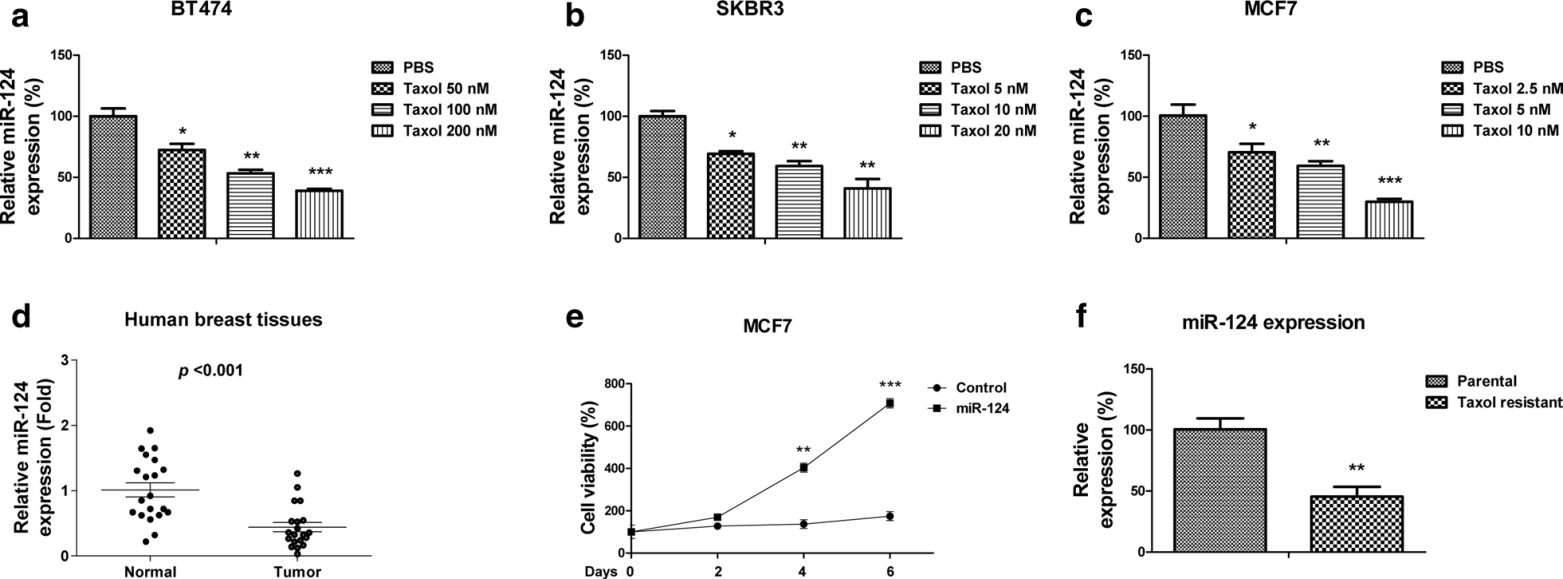
MiR-124 is negatively correlated with Taxol resistance. **a** BT474, **b** SKBR3 and **c** MCF7 cells were treated with or without Taxol at the indicated concentrations for 48 h, the expressions of miR-124 were detected by qRT-PCR. **d** Expressions of miR-124 were detected from human primary breast cancer tissues and their matched adjacent normal breast tissues by qRT-PCR. **e** MCF7 cells were transfected with control miRNAs or miR-124 for 48 h, followed by the measurements of cell viability at 0, 2, 4 and 6 days. **f** Expressions of miR-124 from MCF7 parental cells and Taxol resistant cells were measured by qRT-PCR. *p < 0.05; **p < 0.01; ***p < 0.001

**Fig. 4:**
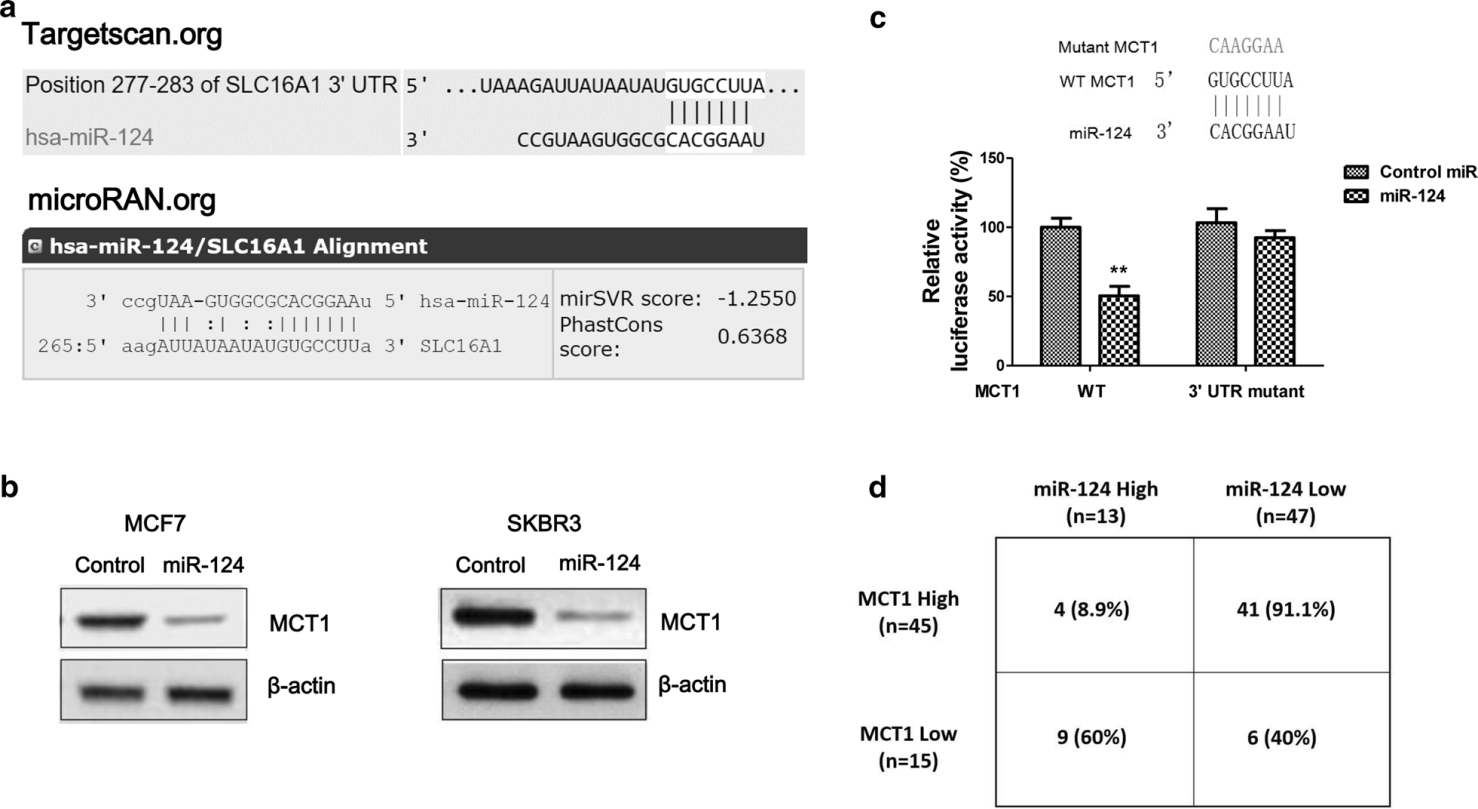
MCT1 is a direct target of miR-124 in breast cancers. **a** Prediction programs from Targetscan and microRNA.org illustrated 3′UTR region of SLC16A1 mRNA contains complimentary binding sites for the seeding sequence of miR-124. **b** Western blot analysis of the downregulated expression levels of MCT1 in miR-124 overexpressed MCF7 (left) and SKBR3 (right) cells. β-actin is a loading control. **c** Luciferase assays showed miR-124 targeted predicted 3′UTR region of MCT1 but not the mutant 3UTR. **d** Analysis of the negative correlation between MCT1 mRNAs and miR-124 expressions in human breast cancer specimens. **p < 0.01

To investigate the mechanisms of the Taxol-mediated lactate homeostasis disorder, we focused on microRNAs which have been known as essentially endogenous regulators of cancer glucose metabolism. We found miR-124 expressions were significantly downregulated by treating with low concentration-Taxol in three breast cancer cells, BT474, SKBR3 and MCF7 (Fig. 3a–c), suggesting miR-124 might involve in the negative feedback of Taxol-MCT1 pathway since miRNAs negatively regulate the expression of their target genes through direct binding to 3′UTR region. Consistent with previous study that miR-124 acts as a tumor suppressive miRNA [16–21], we found miR-124 was downregulated in human breast cancer patient samples compared with their adjacent normal tissues (Fig. 3d). Overexpression of miR-124 suppressed cell growth rates of MCF7 cells (Fig. 3e). To investigate the roles of miR-124 in Taxol sensitivity, we compared the expressions of miR-124 in Taxol resistant cells and parental cells. As we expected, as a potential tumor suppressive miRNA, miR-124 was significantly downregulated in Taxol resistant cells (Fig. 3g). To identify potential targets of miR-124 in breast cancer cells, we searched two online miRNA target prediction algorithms, microRNA.org and TargetScan. Both software predicted MCT1 as a putative target of miR-124. MCT1 contains a conserved 7-mer nucleotide at positions 277–283 on its 3′UTR as a miR-124 binding sites (Fig. 4a). To verify whether miR-124 could target MCT1 in breast cancer cells, we overexpressed miR-124 by transfecting miR-124 precursor into two breast cancer cells. MCF7 and SKBR3 cells displayed significantly downregulated MCT1 expressions (Fig. 4b). To determine whether MCT1 is a direct target of miR-124 through binding to the predicted 3′UTR region, we performed luciferase assay. A fragment of around 300 base pairs of the MCT1 3′UTR containing miR-124 binding sequences downstream of the luciferase coding regions was inserted into luciferase reporter. The constructs containing either WT or mutant 3′UTR of MCT1 were co-transfected with control miR-124 or miR-124 precursor into 293T cells (Fig. 4c). As shown in Fig. 4c, luciferase activity of vector containing WT 3′UTR of MCT1 decreased about 50% by co-transfecting with miR-124 compared with control miRNA. By contrast, the luciferase activities of vector containing mutant 3′UTR of MCT1 are poorly affected by miR-124 co-transfection. These data verified that miR-124 could directly target MCT1 by binding its 3′UTR. To investigate whether miR-124 could target MCT1 in human breast cancer patients, we evaluated the correlation between MCT1 transcript levels and miR-124 expressions in a series of 60 primary breast cancer patient samples by qRT-PCR analysis. Statistical analysis illustrated high percentages of low miR-124 expression in MCT1 high-expressed samples (91.1%), while among the MCT1 low-expressed breast cancer samples, high expressed miR-124 presented a high percentage (60%) (Fig. 4d). Taken together, these results demonstrated miR-124 could directly target MCT1, correlating with the miR-124-mediated Taxol sensitivity.

### The combination of MCT1 inhibition with Taxol exhibits synergistic cytotoxicity on breast cancer cells

**Fig. 5:**
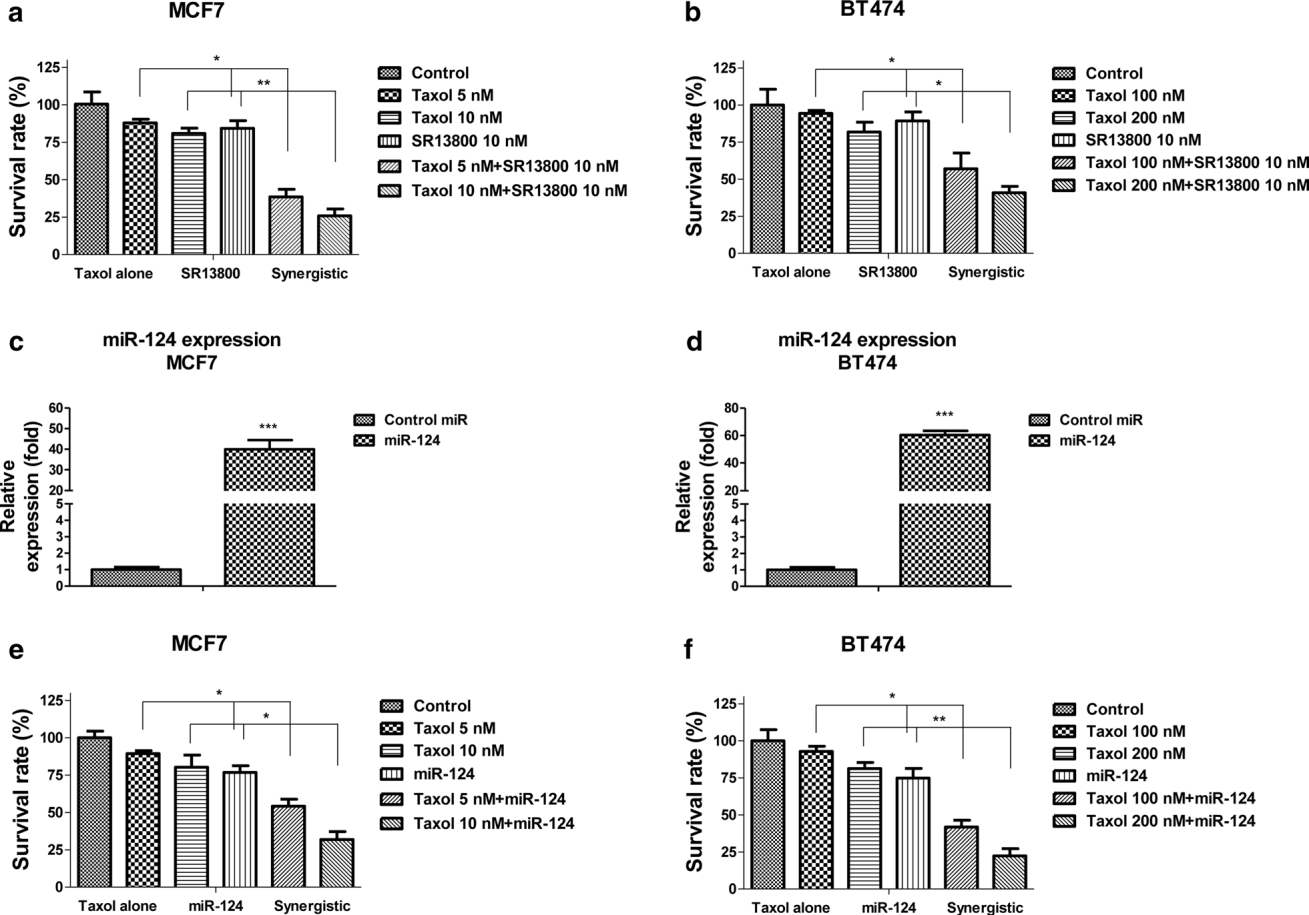
Combination of Taxol and MCT1 inhibition demonstrates synergistic inhibition on breast cancer cells. **a** MCF7 cells and (**b**) BT474 cells were treated with control, Taxol alone, MCT1 inhibitor alone or the combination of Taxol and MCT1 inhibitor at the indicated concentrations for 48 h, the cell viabilities were measured. **c** MCF7 and (**d**) BT474 cells were transfected with control miRNA or miR-124 for 48 h, the expressions of miR-124 were measured by qRT-PCR. **e** MCF7 and (**f**) BT474 cells were treated with control, Taxol alone, miR-124 overexpression or the combination of Taxol and miR-124 at the indicated concentrations for 48 h, the cell viabilities were measured. *p < 0.05; **p < 0.01; ***p < 0.001

Given the high correlation among miR-124, Taxol sensitivity and MCT1 in breast cancers, we wondered whether the combination of MCT1 inhibitor or miR-124 with Taxol could improve the anticancer chemotherapeutic effects. We thus treated MCF-7 and BT474 cells with control, Taxol alone, MCT1 inhibitor alone or the combination of both. Though Taxol or SR13800 alone at low concentrations had little toxic effect, co-treatment of MCF-7 or BT474 cells with Taxol and SR13800 led to significant cell death (Fig. 5a, b). Synergy of Taxol plus SR13800 was evident in these two MCT1 expressing breast cancer cells (Fig. 5a, b). Yet, as expected, no synergistic effects on MCT1 negative human breast cancer cells, MDA-MB-231 (not shown). To test whether the combination of miR-124 and Taxol could achieve synergistic inhibitory effects, we overexpressed miR-124 in MCF7 and BT474 cells (Fig. 5c, d), followed by the co-treatment of breast cancer cells with Taxol plus miR-124. As we expected, synergy of Taxol plus miR-124 was evident in MCF7 and BT474 cells (Fig. 5e, f). Taken together, the above results indicate selective targeting MCT1 could augment the anticancer potency of Taxol.

### Inhibition of MCT1 by miR-124 re-sensitizes Taxol resistant cells through suppression of glycolysis in vitro and in vivo

**Fig. 6:**
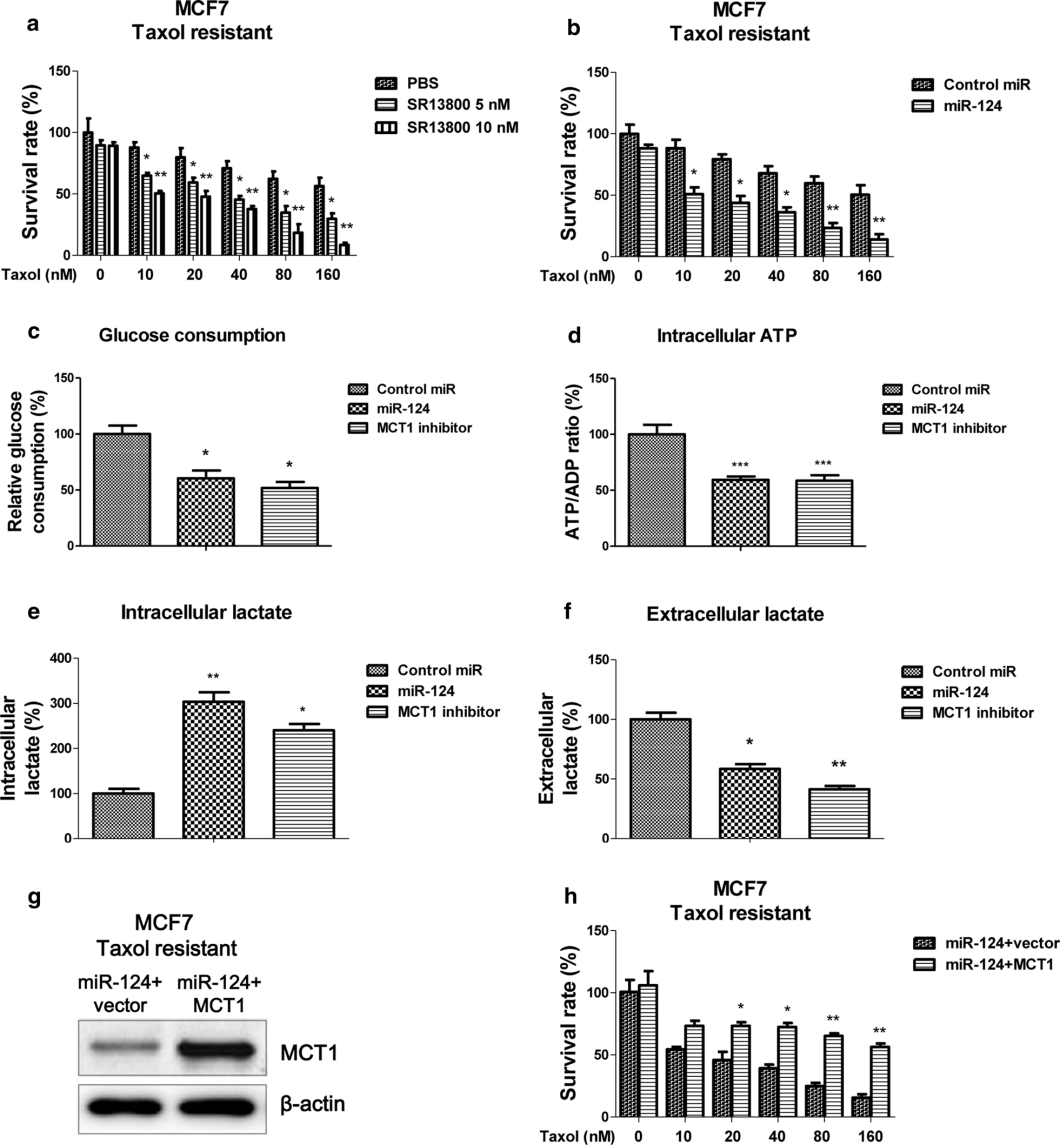
Re-sensitization of Taxol resistant cells by MCT1 inhibition. **a** MCF7 Taxol resistant cells were treated with control, Taxol alone, MCT1 inhibitor alone or the combination of Taxol and MCT1 inhibitor at the indicated concentrations for 48 h, the cell viabilities were measured. **b** MCF7 Taxol resistant cells were transfected with control miRNAs or miR-124 for 48 h, followed by the treatments of Taxol at 0, 10, 20, 40, 80 and 160 nM for 48 h. Cell viabilities were measured by MTT assay. **c** MCF Taxol resistant cells were treated with control, miR-124 or MCT1 inhibitor for 48 h, the glucose uptake, (**d**) cellular ATP/ADP ratio, (**e**) intracellular lactate and (**f**) extracellular lactate were measured. **g** MCF7 Taxol resistant cells were co-transfected with miR-124 plus control plasmid or miR-124 plus MCT1 overexpression plasmid for 48 h, the expressions of MCT1 were detected by Western blot and (H) the cell viabilities were measured under Taxol treatments. β-actin is a loading control. *p < 0.05; **p < 0.01

**Fig. 7:**
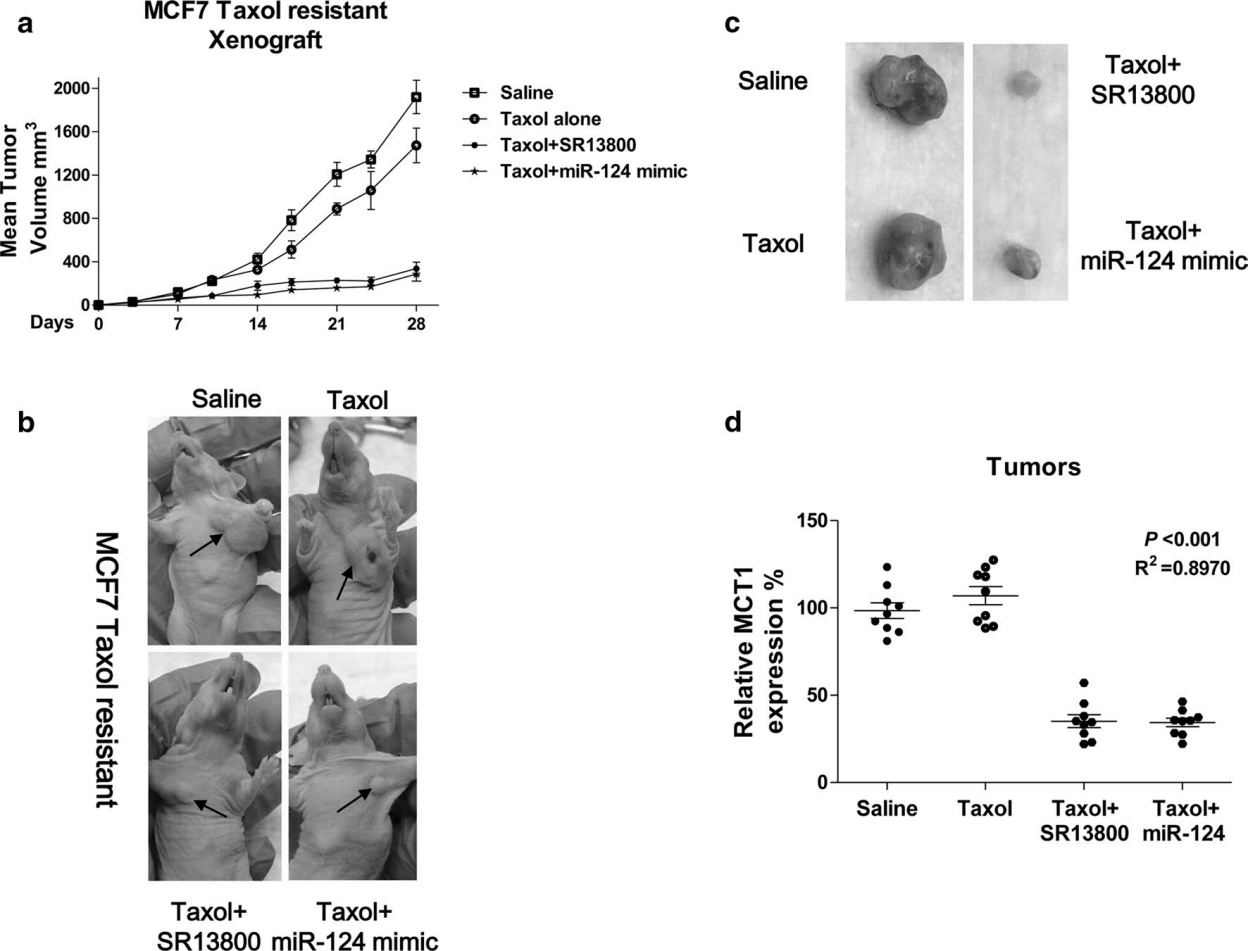
Combination of Taxol and MCT1 inhibition effectively suppresses Taxol resistant tumor growth in vivo. **a**–**c** Comparison of tumor volumes in pre-established MCF7 Taxol resistant cells tumor xenografts under treatments with control, Taxol alone (10 mg/kg, i.p., 3 times/wk), Taxol plus SR13800 (10 mg/kg, i.p., 3 times/wk), or Taxol plus miR-124 mimic (5 µL of 50 µM, i.p., 3 times/wk). **d** MCT1 mRNA expressions in MCF7 Taxol resistant cells tumor xenograft treated with control, Taxol alone, Taxol plus SR13800 and Taxol plus miR-124 mimic. *p < 0.05; **p < 0.01

We demonstrated the negative correlation between miR-124 expression and Taxol sensitivity in breast cancer cells (Fig. 3). To assess whether overexpression of miR-124 could sensitize Taxol resistant cells, we co-treated MCF7 Taxol resistant cells with Taxol and MCT1 inhibitor. Results in Fig. 6a demonstrated Taxol resistant cells displayed significant cell death rate under the Taxol (10, 20, 40, 60 and 80 nM) plus SR13800 (5 or 10 nM). Similar results were observed in MCF7 Taxol resistant cell with overexpression of miR-124 (Fig. 6b), suggesting targeting MCT1 maybe an effect approach against Taxol resistance. Furthermore, Taxol resistant cells had decreased levels of glucose consumption (Fig. 6c) and intracellular ATP (Fig. 6d), increased intracellular lactate accumulation (Fig. 6e) and decreased lactate exportation (Fig. 6f) under either overexpression of miR-124 or MCT1 inhibitor treatments. These findings present a strong correlation between Taxol sensitivity and the MCT1-mediated lactate secretion. To verify whether the miR-124-modulated Taxol sensitivity was through MCT1 inhibition, we performed rescue experiments by endogenously overexpression of MCT1 into miR-124-overexpressing Taxol resistant cells (Fig. 6g). Notably, restoration of MCT1 significantly promoted the survival rates of miR-124-overexpressing Taxol resistant cells under 20, 40, 80 or 180 nM Taxol treatments (Fig. 6h). Therefore, in vitro rescue experiments demonstrated sensitization of Taxol resistant cells by miR-124 was through inhibiting MCT1. To examine whether inhibiting MCT1 impacts Taxol sensitivity of breast cancer tumors in vivo, we performed xenograft experiments by injection of MCF7 Taxol resistant cells into nude mice and began Taxol alone, Taxol plus MCT1 inhibitor or Taxol plus miR-124 mimic treatment after the tumors reached 5 mm in diameter. The combination of Taxol with either SR13800 or miR-124 mimic robustly suppressed growth of the mammary fat pad xenograft tumors (Fig. 7a). The tumor sizes of the combined treatments were significantly smaller than control or Taxol alone treatment (Fig. 7b, c). Moreover, qPCR results illustrated the MCT1 mRNA levels were significantly downregulated by miR-124 mimic or SR13800 treatments compared with control or Taxol alone treatment (Fig. 7d). These data are consistent with our in vitro results showing that miR-124 sensitizes Taxol resistant breast cancer cells through MCT1 inhibition.

## Discussion

It was known that increased aerobic glycolysis is a hallmark of cancer cells [6]. Glucose consumption and glycolytic flux is augmented, leading to the production of intermediates necessary for fast growth of cancer cells [6]. Furthermore, mounting evidence attributes the chemoresistance to dysregulated cellular metabolism [25]. In this study, we demonstrated low dose Taxol treatments induced glycolysis flux of breast cancer cells. Consequently, Taxol resistant cells displayed upregulated glucose consumption and lactate production, suggesting targeting glycolysis may result in augments of chemotherapeutic effects.

Under stress, metabolic reprogramming provides cancer cells survival advantage, presenting one of the hallmarks of cancer. Recent studies and our results demonstrated MCT1 is upregulated in human malignancies [11], suggesting an oncogenic role of MCT1. Our findings showed that inhibition of MCT1 by either specific siRNA knockdown or inhibitor significantly suppressed glycolysis and lactate homeostasis of breast cancer cells. In addition, we report inhibition of MCT1 led to a metabolic shift from anaerobic glycolysis to oxidative phosphorylation, a revered “Warburg effects”. These findings are consistent with previous studies showing another MCT1 inhibitor, AZD3965 could reduce glycolysis and proliferation rates but increases oxygen consumption [26]. In addition to their discoveries, we firstly demonstrated MCT1 inhibition by either siRNA knockdown or inhibitor treatment sensitizes Taxol resistant breast cancer cell. In our mammary fat pad xenograft model, Taxol plus MCT1 inhibition treatment resensitized Taxol resistant tumor growth. Our in vitro and in vivo results consistently showed synergistic antitumor effects with the combined treatments of Taxol and MCT1 inhibition, providing new approach for anti-chemoresistance of breast cancer. However, the mechanism by which blocking MCT1 impacts Taxol sensitivity remains unclear. We showed inhibition of MCT1 led to accumulation of intracellular lactate, which may trigger endogenous toxicity of breast cancer cells. These results are consistent with previous study [12]. In addition, regulation of cellular redox status, disruption of intracellular pH homeostasis, or bioenergetics pathway could be possible mechanisms.

Studies indicate that miR-124 plays a tumor suppressive role in malignant progression of human cancers [16–21]. It has been reported that miR-124 inhibits proliferation in cancer cells through targeting CDK4 [21], CBL [27], Slug [28] and androgen receptor [29]. We firstly demonstrated MCT1 as a direct target of miR-124 in breast cancer cells and patient specimens. MiR-124 directly binds the 3′-UTR of MCT1. Notably, miR-124 was downregulated in Taxol resistant cells compared with parental cells, consistent with previous description that miR-124 has tumor suppressive functions. Furthermore, rescue experiments supported overexpression of miR-124 suppressed proliferation rates and increased Taxol sensitivity of breast cancer cells through targeting MCT1, indicating miR-124 may serve as an ideal therapeutic target against chemoresistant breast cancer.

## Conclusion

In summary, our study reveals a miR-124-MCT1-Taxol sensitivity axis, providing a new mechanism for the miRNA-mediated Taxol sensitivity. These findings may provide insight into the identification of novel therapeutic targets against chemoresistant breast cancers.

## Data Availability

Please contact author for data requests.

## Abbreviations

MCT1: monocarboxylate transporter 1
LDHA: lactate dehydrogenase-A
miRNAs: microRNAs
UTR: untranslated region
OSCC: oral squamous cell carcinoma
HCC: hepatocellular carcinoma
DMSO: dimethyl sulfoxide
IACUC: Institute Animal Care and Use Committee
